# *In Vitro* Characterization of Human Mesenchymal Stem Cells Isolated from Different Tissues with a Potential to Promote Complex Bone Regeneration

**DOI:** 10.1155/2016/3595941

**Published:** 2016-11-24

**Authors:** Áron Szepesi, Zsolt Matula, Anna Szigeti, György Várady, József Szalma, Gyula Szabó, Ferenc Uher, Balázs Sarkadi, Katalin Német

**Affiliations:** ^1^Research Centre for Natural Sciences, Hungarian Academy of Sciences, Budapest, Hungary; ^2^Creative Cell Ltd., Budapest, Hungary; ^3^Department of Dentistry, Oral and Maxillofacial Surgery, University of Pécs Medical School, Pécs, Hungary; ^4^National Blood Service, Budapest, Hungary; ^5^Molecular Biophysics Research Group of the Hungarian Academy of Sciences, Semmelweis University, Budapest, Hungary

## Abstract

Bone tissue regeneration is a major, worldwide medical need, and several strategies have been developed to support the regeneration of extensive bone defects, including stem cell based bone grafts. In addition to the application of stem cells with high osteogenic potential, it is important to maintain proper blood flow in a bone graft to avoid inner graft necrosis. Mesenchymal stem cells (MSCs) may form both osteocytes and endothelial cells; therefore we examined the combined* in vitro* osteogenic and endothelial differentiation capacities of MSCs derived from adipose tissue, Wharton's jelly, and periodontal ligament. Based on a detailed characterization presented here, MSCs isolated from adipose tissue and periodontal ligament may be most appropriate for generating vascularized bone grafts.

## 1. Introduction

There is a great clinical demand for improved methods of bone tissue regeneration after trauma or medical bone resections, as spontaneous recovery is slow or in many cases nonexistent. This is well exemplified in critical-size bone defects [1], which may affect the oral cavity as a result of trauma, aggressive periodontitis, or tumor surgery [2]. Since the availability of donor-derived bone grafts is limited and the extraction of autologous bone grafts is often associated with considerable pain and donor-site morbidity, there is an increasing demand for* in vitro* engineered bone tissues [3].

In order to help the bone healing process, recent technologies promote the use of various tissue grafts, containing autologous or allogenic bone-forming cells. The cells used in such a bone tissue regeneration therapy should have high bone-forming potential for rapid and effective incorporation.

In addition to osteoblasts, cells promoting angiogenesis should be also important participants in bone graft formation and regeneration, especially in the case of large bone defects, in order to promote osseointegration and avoid inner graft necrosis. Clearly, to ensure the long-term function of the bone graft, cells capable of forming blood vessels within the bone tissue should also be present [4]. Stem or progenitor cells capable of differentiating into both osteoblast and blood vessel endothelial cell may provide a solution in this regard.

Mesenchymal stem cells (MSCs) are currently considered to be key sources in regenerative medicine. MSCs are multipotent with self-renewal ability and a capacity to differentiate into different mesenchymal lineages, including adipose tissue, bone, and cartilage [5, 6]. Tissue-derived MSCs do not raise major ethical concerns in medical applications [7]. In addition, due to their immunosuppressive characteristics even allogeneic MSCs can be used in various therapeutic approaches [8].

MSCs were initially described in the bone marrow stroma [9] and later found in numerous human tissues including fat and in different dental tissues [5, 10]. Bone marrow derived (BM) MSCs have been used for tissue engineering for several years. However, the invasive collection procedure of BM-MSCs possesses disadvantages, including pain and potential medical complications [5]. MSCs from adult adipose tissue (AD-MSCs) can be readily harvested in large numbers with very low donor-site morbidity. AD-MSCs therefore are broadly used in various regenerative therapeutic interventions and may also serve as cells for bone grafts [11]. In addition Wharton's jelly of the umbilical cord (WJ-MSC) and the tissues of the placenta also provide a rich source of MSCs for potential therapies [12].

The periodontal ligament is a soft connective tissue developed from the neural crest and has anchoring, homeostatic, and regenerative functions in the periodontium. Periodontal ligament contains stem cells (PDLSC) possessing MSC properties [10, 13]. The enhanced osseointegration effect of human PDLSCs was confirmed after transplantation of these cells with bone graft [14].

In the present work we have compared several properties of* in vitro* cultured and differentiated human AD-MSCs, WJ-MSCs, and PDLSCs. Under identical conditions we have examined their frequency of colonies, immunophenotype, trilineage differentiation capacity, and the expressed pluripotency and mesodermal markers. Furthermore, we have characterized in detail the endothelial differentiation capacity of these MSCs.

## 2. Materials and Methods

### 2.1. Isolation, Culturing, and Differentiation of Cells

All samples were obtained from healthy donors. Work with human tissues was performed with the permission of the Ethical Committee of the Hungarian Medical Research Council (ETT; ID: 24083-3/2013/HER). Most of the reagents below were purchased from Thermo Fisher Scientific (Waltham, USA), all others as indicated.

**Figure 1:**
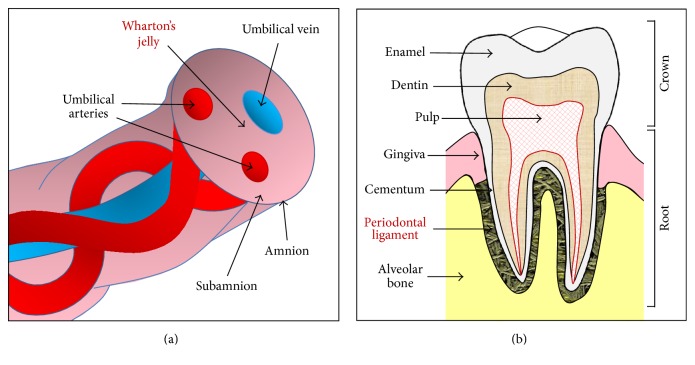
Schematic cross-sectional diagram of human umbilical cord (a) and tooth (b) shows anatomical compartments, including Wharton's jelly (a) and periodontal ligament (b), as a source of stem cells.

MSCs from liposuction derived adipose tissue (*n* = 3) and periodontal ligament (*n* = 3) were isolated as described previously [13, 15]. Wharton's jelly of the umbilical cord was collected from full-term births (*n* = 3). The anatomical localization of the periodontal ligament and Wharton's jelly is presented on Figure 1.

For isolation of Wharton's jelly derived MSC, the cord was cut into 1-2 cm segments and the vessels were removed. The outstretched tissue was treated with 2 mg/mL collagenase type IV and 100 IU/mL hyaluronidase (Sigma-Aldrich, St. Louis, USA). The cord pieces were washed with DMEM medium and Wharton's jelly was collected and passed through an 18 G needle. The cell suspension was then resuspended in high glucose DMEM medium supplemented with 20% fetal bovine serum (FBS) and 16 ng/mL fibroblast growth factor 2. Cells were plated with 2 × 10^5^/cm^2^ density. Growth medium (DMEM-F12 1 : 1 with 10% FBS, 2 mM L-glutamine, and 50 *μ*g/mL gentamicin plus 1 ng/mL FGF-2) was used after third medium changes, and cells were subcultured once a week at a density of 4 × 10^3^/cm^2^.

To assess the differentiation potential of the MSC isolates, cells between passages 4 and 8 were differentiated into adipocyte, osteoblast, and chondrocyte phenotypes as documented [13].

Human umbilical vein endothelial cells (HUVEC) were kind gifts of Adrienn Németh. The isolation method and culture conditions were detailed by [16].

The human embryonic stem cell line 9 (HUES9) was a kind gift of Douglas Melton, Harvard University, USA. The HUES culture conditions were used as described in detail by Apáti et al. [17].

### 2.2. Flow Cytometry

The expression of cell surface immunomarkers was assessed by flow cytometry [13]. Cells were stained with the following mouse monoclonal antibodies: anti-human CD13, CD14, CD29, CD31, CD34, CD44, CD45, CD73, CD90, CD105, CD106, CD117, CD133, CD144, CD146, CD166, CD271, CD309, and HLA-DR or the corresponding isotype controls. For references of the antibodies see Supplementary Table 1 in Supplementary Material available online at http://dx.doi.org/10.1155/2016/3595941. Dead cells were identified by staining with propidium iodide. Measurements were carried out in a 4-color FACSCalibur flow cytometer (BD Biosciences). In the case of ABCG2, unconjugated 5D3 monoclonal antibody was used as described previously [13].

Data were expressed both as mean percentage of positive cells and as median fluorescence intensity (MFI) ratio determined using the median fluorescence value of the specific marker analyzed, divided by the median fluorescence value of the isotype control.

### 2.3. Colony Forming Unit-Fibroblast Assay (CFU-F)

The CFU-F assay was performed at passages 3–6 as previously described [13].

### 2.4. Immunocytochemistry

Cells were fixed [15] and stained with unconjugated monoclonal anti-*α*-smooth muscle actin (*α*-SMA; Sigma-Aldrich; cat. number: A5228) or anti-GATA6 (R&D Systems Minneapolis, USA; cat. number: AF1700) primary antibody or the matched isotype control for 1 hour at room temperature in the dilution according to manufacturer's instructions. AlexaFluor488 (*α*-SMA) or AlexaFluor568 (GATA6) secondary antibodies were applied for fluorescent detection, and nuclei were counterstained with DAPI. The fluorescence membrane stain DiSC_3_(5) was used in final concentration of 5 *μ*M.

### 2.5. Endothelial Differentiation and Matrigel Tube Formation Assay

Cells were stimulated with Endothelial Cell Growth Medium (EGM-2, Lonza, Basel, Switzerland) for 7 days in a humidified thermostat. Chilled Matrigel (Corning, New York, USA) was added to a 24-well plate and incubated at 37°C. 5 × 10^4^ of the induced cells were suspended in EGM-2 medium and added to the solidified Matrigel. Morphological changes were observed under an inverted microscope during a 24-hour incubation period. Representative pictures were taken in every hour and analyzed using the TubeCount software [18] to determine the number and length of tubes. When a maximum level of tube forming was observed, three pictures were taken from different fields of each well. The data were presented as averages of three different experiments.

### 2.6. Gene Expression Analysis

Total RNA was isolated from undifferentiated and differentiated cells and cDNA was synthesized [15]. The expression levels of OCT4, SOX2, NANOG, telomerase reverse transcriptase (TERT), tissue-nonspecific alkaline phosphatase (ALP), peroxisome proliferator-activated receptor gamma (PPAR*γ*), and platelet endothelial cell adhesion molecule 1 (PECAM1) genes were measured using TaqMan reagents. The expression of runt-related transcription factor 2 (RUNX2) mRNA was determined by using Power SYBR Green reagents in StepOne Plus qPCR instruments (Thermo Fisher Scientific). Gene expression levels were calculated with 2^(−ΔCt)^ method relative to glyceraldehyde-3-phosphate dehydrogenase (GAPDH) as a reference. For references of the primers see Supplementary Table 2.

### 2.7. Statistical Analysis

The data are presented as the mean of three repeated experiments of the biological parallels ± SD. Statistical comparisons were performed using Student's* t-test*. *P* values <0.001 were considered significant.

## 3. Results

### 3.1. Colony Forming Unit-Fibroblast Assay

**Figure 2:**
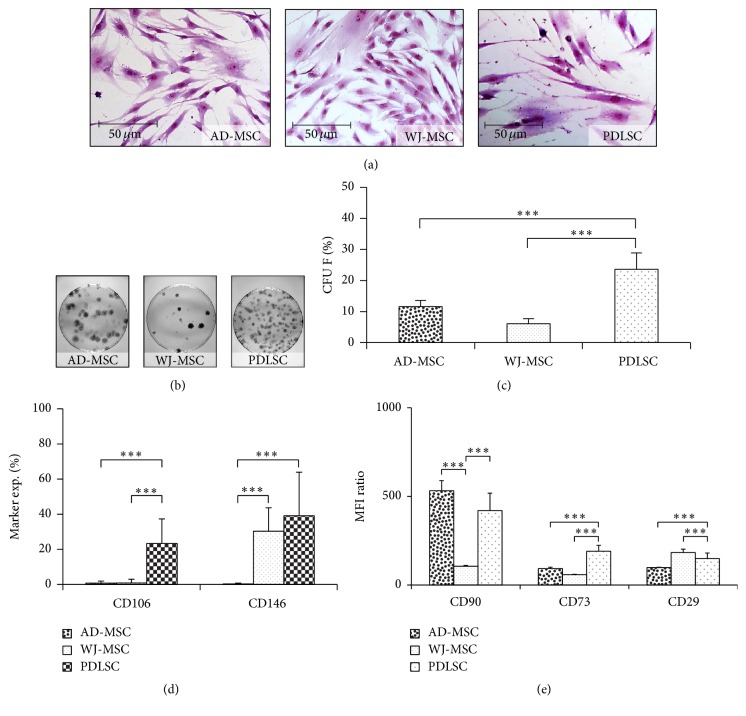
Heterogeneity of the different MSC samples. The morphology of the cells was demonstrated with Giemsa staining (a). The colony forming ability of MSCs was investigated on 10 cm petri dishes (b). Colony forming efficiency was calculated as the number of colonies divided by the total number of seeded cells (c). The CD106 and CD146 expression in different MSC isolates determined by flow cytometry (d). The expression levels of CD90, CD73, and CD29 markers of MSC samples were determined with flow cytometry and represented as median fluorescence intensities (MFI) (e). The photos demonstrate representative samples. ^*∗∗∗*^ *P* value less than 0.001.

The efficiency by which MSCs can form colonies at low densities on the plastic surface still remains an important assay for the quality control of cell preparations. (The heterogeneity of the different MSC isolates is demonstrated in Figure 2(a).) PDLSCs produced 94.3 ± 27.0 colonies from 400 cells which is significantly higher than in the case of AD-MSC (46.3 ± 21.0) or WJ-MSC (24.2 ± 8.9) isolates (Figures 2(b) and 2(c)).

### 3.2. Cell Surface Markers

The immunophenotypic profile of adherent cells from each culture was determined by testing a panel of surface markers using flow cytometry. Essentially, 100% of all the different MSCs expressed the most commonly reported positive markers CD13, CD29, CD44, CD73, CD90, CD105, and CD166. Endothelial markers CD31, CD144, and CD309 and the markers involved in hematopoiesis like CD14, CD34, CD45, CD117, or CD133 and HLA-DR were absent or indeterminably low. It should be noted that CD90 expression decreased during passages in the case of a WJ-MSC isolate.

In agreement with the data in the literature, the vascular cell adhesion molecule 1 (VCAM-1, CD106) was present in a small subpopulation of the PDL (20.6 ± 4.5%) derived cells, while AD-MSCs and WJ-MSCs failed to express this marker. The melanoma cell adhesion molecule (MCAM, CD146), known as a pericyte marker, was present in 25–55% of the WJ-MSC and PDL cells, while AD-MSCs, in contrast to data in the literature, did not express CD146 (Figure 2(d)).

The common MSC markers were found to be present in all cell types examined, while marker density was variable in the MSCs from different tissue sources. The presence or the expression level of a marker in MSCs usually refers to a special property of the cell. For example, it has been described that CD29, CD44, CD73, CD90, CD105, and CD166 have roles in the multilineage differentiation processes [19–21]. Thus we tried to establish a correlation between the expression levels of these surface markers and the osteogenic, adipogenic, or angiogenic differentiation potential of the MSC isolates from different tissue sources. The expression intensity of the markers was determined by flow cytometry. In our experiments we found that Thy-1 (CD90) was present at high levels on the surface of AD-MSCs and PDLSCs, while this marker level was low in the case of WJ-MSCs. The ectonucleotidase CD73 was expressed at significantly higher levels in the PDL cells, as compared to other MSC isolates. Furthermore, the integrin family member CD29 was expressed only modestly in AD-MSCs (Figure 2(e)).

### 3.3. Osteogenic, Adipogenic, and Chondrogenic Differentiation

**Figure 3:**
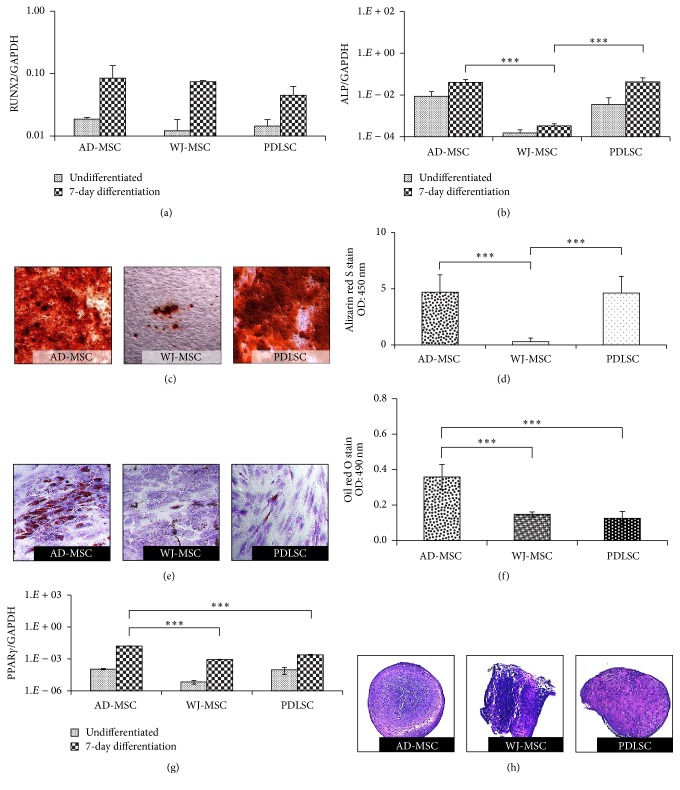
The trilineage differentiation ability of MSC isolates. The expression level of the RUNX2 (a) and ALP (b) genes in the undifferentiated and differentiated samples, determined with RT-qPCR measurements. Alizarin red staining was used to demonstrate the calcium deposition of the matured bone matrix after 14 days of osteogenic induction (c). The stain was dissolved and quantified colorimetrically (d). Lipid accumulation was confirmed by oil red O staining (red dots) after adipogenic induction; cells were counterstained with dimethyl methylene blue stain (e). The concentration of the red stain was determined colorimetrically (f). The adipogenesis was followed with RT-qPCR measurement of PPAR*γ* gene (g). Chondrogenic differentiation was confirmed in all samples (h). The photos demonstrate representative samples. ^*∗∗∗*^ *P* value less than 0.001.

The trilineage differentiation capacity of MSCs was confirmed by standard induction protocols. The bone-forming ability of MSCs was followed by RT-qPCR analysis of RUNX2 and ALP mRNA expression after 7 days of differentiation in an osteogenic medium. RUNX2 is the master transcription factor in osteogenic development, while ALP is recognized as an early marker of osteoblastic differentiation and indispensable for extracellular matrix maturation. When induced, all cell types showed upregulated expression of RUNX2, and no significant differences were noticeable between MSC isolates in this expression (Figure 3(a)). After stimulation, the increased expression levels of ALP mRNA were found to be similar in AD-MSCs and PDLSCs, while significantly lower ALP gene expression was observed in the WJ-MSC samples (Figure 3(b)).

After 14 days of osteogenic MSC differentiation induction, alizarin red staining was performed to directly visualize the mineralization of the extracellular matrix in these cultures. The quantification of the staining showed the high calcium accumulation in AD-MSC and PDLSC isolates. The mineralization capability of WJ-MSCs lagged far behind those observed in MSCs from other tissues (Figures 3(c) and 3(d)). We observed significant correlation between the CD90 expression patterns and the levels of calcium deposition in the different MSC isolates (Figure 2(e)).

In the adipogenic differentiation experiments AD-MSCs accumulated significantly higher amounts of lipids, as compared to the other MSC preparations (Figures 3(e) and 3(f)). The mRNA expression levels of PPAR*γ*, a key factor in adipogenesis, were in close correlation with the amounts of lipid deposition, indicating that AD-MSCs can produce adipocytes most effectively (Figure 3(g)). We found that CD29 expression levels in AD-MSCs were the lowest (Figure 2(e)); thus CD29 expression may be inversely related to the adipogenic differentiation potential as in accordance with the findings of Rodeheffer et al. [22].

We have also performed chondrogenic differentiation of the various MSC preparations. We found that all MSC types were capable for this differentiation (Figure 3(h)).

### 3.4. Endothelial Differentiation

**Figure 4:**
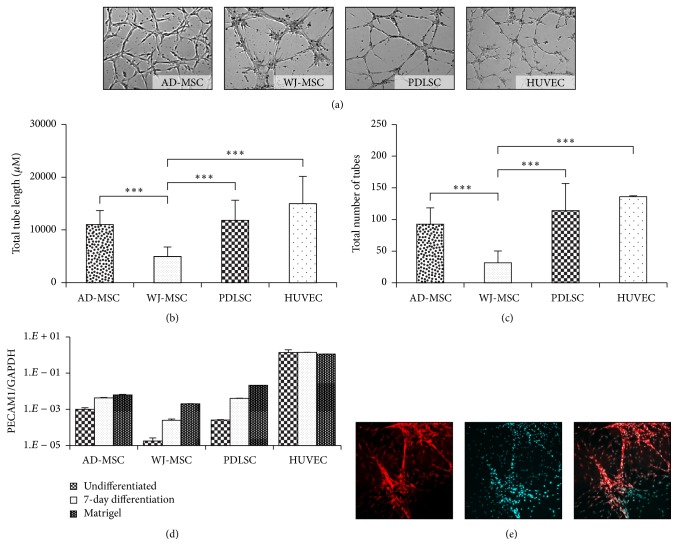
Endothelial differentiation potential of the MSCs was tested in Matrigel tube formation assay. Tubular-like structures were photographed (a) and analyzed with TubeCount software. Human umbilical vein endothelial cells (HUVEC) were used as controls. Total tube length (b) and numbers (c) were counted and presented in the diagrams. The changes in the expression of PECAM1 during endothelial differentiation were followed with RT-qPCR. Samples were collected from undifferentiated and predifferentiated states (7-day endothelial induction) and after angiogenesis in a Matrigel assay (d). The 3D network developed by a representative PDLSC sample was demonstrated with fluorescence staining of the cell membrane by DiSC_3_(5) (red) and the nucleus by DAPI (cyan) (e). The photos demonstrate representative samples. ^*∗∗∗*^ *P* value less than 0.001.

The endothelial forming potential of the MSCs was examined in an angiogenesis assay, wherein we found that AD-MSCs and WJ-MSCs reached a maximum tube-forming potential between 3 and 7 hours after seeding, while the PDLSCs needed 18–20 hours to form a well-developed capillary-like tubule network. The tube-forming potential of AD-MSCs and PDLSCs was similar to that observed in the HUVEC, while the tubular networks formed by WJ-MSCs were significantly less developed, as compared to other isolates (Figures 4(a), 4(b), and 4(c)). Moreover, the tubes generated by MSCs derived from different tissues showed significantly different phenotypes. AD-MSCs and WJ-MSCs formed relatively short tubes and the tubes of WJ-MSCs were much thicker than those formed by the other MSC isolates. The extensive tubular network formed by PDLSC mostly consisted of long and thin tubes, resembling capillaries. A characteristic sample of a tubular network formed by fluorescently stained PDL stem cells is demonstrated in Figure 4(e).

The messenger RNA expression of the endothelial specific marker PECAM1 was analyzed by real-time qPCR after prestimulation of the MSCs and after retrieval of the cells from the Matrigel (Figure 4(d)). An elevation in PECAM1 mRNA expression level was observed after the induction in all MSC preparations. Furthermore, an additional elevation was noticeable in the expression of PECAM1 when the cells formed endothelial structures. However, PECAM1 mRNA expression was still significantly smaller as compared to that found in the HUVEC, in which no further activation of this gene expression was observed either in an angiogenesis inducing medium or in the Matrigel environment.

All these results indicate a higher endothelial differentiation ability and vessel-forming potential of PDLSCs and AD-MSCs, as compared to the WJ-MSCs.

### 3.5. Expression of Pluripotency and Mesodermal Markers in the MSC Preparations

**Figure 5:**
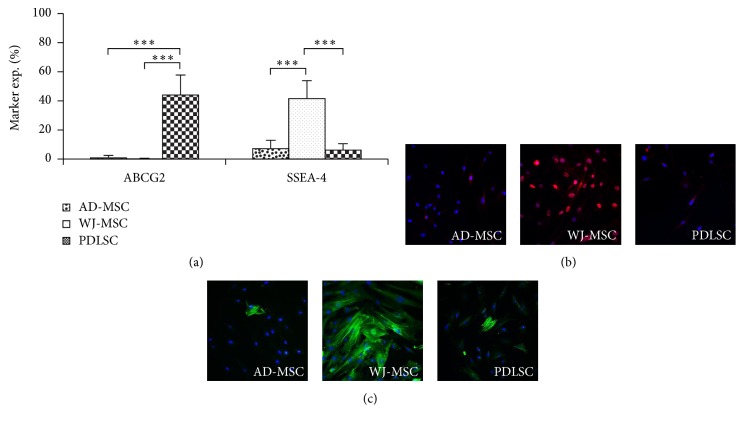
Pluripotency and mesodermal markers of MSCs. The embryonic stem cell marker ABCG2 was detectable uniquely in PDLSCs, while a remarkable SSEA4 expressing population was found in WJ-MSC isolates, as determined by flow cytometer (a). Immunohistochemical staining of GATA6 (red, DAPI-blue) (b) and *α*-SMA (green, DAPI-blue) (c) showed significant expression only in WJ-MSCs. The photos demonstrate representative samples. ^*∗∗∗*^ *P* value less than 0.001.

In these experiments we have examined whether WJ-MSCs, suggested to be in more early stages of stem cell development, express any special pluripotency markers. The expression of mRNA of transcription factors indicating a pluripotent stem cell state, including OCT4, SOX2, Nanog, or that of TERT, a major factor in maintaining self-renewal capacity, was not detectable in either of the MSC isolates examined here (data not shown). However, the stage-specific embryonic antigene-4 (SSEA-4), a marker of undifferentiated state in human embryonic stem cells, was present in a remarkable quantity on the surface of the WJ-MSCs (37–65%), in contrast to that found in AD-MSCs and PDLSCs (6–8%). The presence of the ABCG2 protein, another potential stem cell marker, was detectable with flow cytometry exclusively in the PDLSCs (Figure 5(a)).

In immunocytochemistry studies the mesodermal marker proteins, *α*-SMA, typically expressed in smooth muscle cells, and the transcription factor GATA6, were observable only in WJ-MSCs (Figures 5(b) and 5(c)).

## 4. Discussion

MSCs derived from diverse tissues share some common properties [6]; however, on closer examination, cells with very heterogeneous properties can be found within individual isolates in the expression of various markers. The colony forming capacity and the differentiation potential of these isolates can vary as well.

In this study we found that the expression levels of CD90, CD73, and CD29 were variable, but representative for the MSCs isolated from different tissue sources. Here we observed that AD-MSCs and PDLSCs expressed higher CD90 levels and showed greater bone and endothelial differentiation ability, as compared to WJ-MSCs. In the literature an enhanced bone regeneration potential of AD-MSCs with high level expression of Thy-1 has already been found [23]. These data suggest that MSCs with high CD90 expression may have the ability to differentiate into both bone and endothelial cells.

The use of MSCs for tissue engineering applications was found to be safe [24]. The most relevant question in bone graft tissue engineering is how efficiently a progenitor cell can form bone nodules. In our experiments the calcification potential of AD-MSCs was similar to PDLSCs. However, the osteogenic capacity of WJ-MSCs was significantly lower, as compared to MSCs from other tissue sources. This observation is in agreement with the results of Yu et al. [25], reporting that differentiated PDLSCs showed a better mineral deposition as compared to WJ-MSCs, although no significant differences were observed in the upregulation of RUNX2 gene expression in these MSCs. The elevation in RUNX2 mRNA expression indicates the beginning of the ossification process, while in this period no matured osteoblasts can be developed as yet, as suggested by a low level ALP expression, which is an important factor in matrix maturation [26]. The high RUNX2 and low ALP mRNA levels in the WJ-MSCs may explain the relatively low calcification potential of these cells.

Here we showed that none of the examined MSCs expressed the classical embryonic stem cell markers OCT4, SOX2, hTERT, or Nanog. ABCG2, a potential pluripotency marker, was uniquely detectable at a relative high level on the surface of PDLSCs. The findings that WJ-MSCs expressed the highest level of the pluripotency marker SSEA-4 and only WJ-MSCs expressed *α*-SMA and GATA6 protein altogether indicate that this cell type is at an earlier stage of stem cell development. It may also explain the need for a longer induction time in* in vitro* differentiation experiments [27]. It is in accordance with the findings of Kubo et al. [28], whereas the knockdown of GATA6 expression by siRNA suppressed the self-renewal capacity of human MSCs. The limited differentiation potential of *α*-SMA expressing MSCs has already been reported [29]; however the role of SSEA-4 in the maintenance of pluripotency is still controversial [30, 31].

It is important to find the most suitable cell types and techniques to maintain proper blood flow inside of large bone grafts [5]. MSC therapy has been considered as an alternative clinical intervention. On one hand MSCs help healing by support angiogenesis, mediated by paracrine factors (e.g., VEGF). On the other hand, the endothelial differentiation ability of the transplanted MSCs directly leads to vessel formation. It has been demonstrated in clinical studies that transplantation of allogenic MSCs to the affected area is a safe and efficient procedure to promote regenerative vascularization [32]. Furthermore, the angiogenic potential of dental pulp derived stem cells has already been observed [33], indicating that dental stem cells can be suitable candidates to achieve complex bone replacement. Here we show that PDLSCs can form endothelial structures as effectively as AD-MSCs and thus may generate proper vasculature in bone grafts.

The main therapeutic advantage of AD-MSCs, namely, their availability in large numbers, may be overcome by the faster proliferation rates of PDLSCs (data not shown), while a disadvantage of these latter MSCs is the need for their* ex vivo* culturing to reach the required cell number for therapeutic applications.

As a summary, AD-MSCs and PDLSCs seem to be both suitable for complex bone replacement applications, complemented with a vascular network formed by the same donor cell types. These detailed* in vitro* studies may significantly promote further use of MSCs in* in vivo* therapeutic approaches in bone regeneration.

## Supplementary Material

Supplementary Table 1: The list of antibodies used in flow cytometry experiments.
Supplementary Table 2: The list of primers used in RT-qPCR experiments.
