# Supplementary material for: In Vitro Characterization of Human Mesenchymal Stem Cells Isolated from Different Tissues with a Potential to Promote Complex Bone Regeneration
Source: Stem Cells Int. 2016 Nov 24;2016:3595941. doi: 10.1155/2016/3595941 (PMC5143785; doi:10.1155/2016/3595941)
Supplement: Supplementary file 1 — Supplementary Table 1: The list of antibodies used in flow cytometry experiments. Supplementary Table 2: The list of primers used in RT-qPCR experiments. [file 3595941.f1.pdf]

Supplementary materials

Antigene	Conjugated flourophore	Isotype	Manufacturer	Cat. num.
ABCG2	-	IgG2b	Beckton Dickinson	552823
CD14	FITC	IgG2a	Beckman Coulter	IM0645U
CD29	APC	IgG1	BD Pharmingen	561794
CD31	FITC	IgG1	BD Pharmingen	560984
CD34	FITC	IgG1	Beckton Dickinson	345801
CD44	FITC	IgG2b	BD Pharmingen	560977
CD45	FITC	IgG1	Beckman Coulter	IM0782
CD73	PE	IgG2	BD Pharmingen	550257
CD73	PE	IgG3	BD Pharmingen	550257
CD90	APC	IgG1	BD	559869
CD105	PE	IgG1	BD Pharmingen	560839
CD106	PE	IgG1	BD Pharmingen	561679
CD117	PE	IgG1	BD Pharmingen	555714
CD133	APC	IgG1	Miltenyi biotec	130-090-8226
CD144	APC	IgG1	eBioscience	17-1449-73
CD146	PE	IgG1	BD Pharmingen	561013
CD166	PE	IgG1	BD Pharmingen	559263
CD271	AlexaFluor® 647	IgG1	BD Pharmingen	560877
CD309	PE	IgG1	BD Pharmingen	560872
HLA-DR	PE	IgG2a	BD Pharmingen	560943
SSEA-4	PE	IgG3	R&D	FAB1435P
**Isotype controls**				
IgG1	-	IgG1	Thermo Fisher Scientific	554121
IgG1	FITC	IgG1	Beckton Dickinson	345815
IgG1	PE	IgG1	Beckton Dickinson	345816
IgG1	APC	IgG1	Beckton Dickinson	345818
IgG1	AlexaFluor® 647	IgG1	Beckton Dickinson	557783
IgG2a	-	IgG2a	Thermo Fisher Scientific	559319
IgG2a	FITC	IgG2a	Thermo Fisher Scientific	556652
IgG2a	PE	IgG2a	BD Pharmingen	559319
IgG2b	-	IgG2b	Thermo Fisher Scientific	555740
IgG2b	FITC	IgG2b	BD Pharmingen	555742
IgG3	PE	IgG3	R&D	IC007P

Supplementary Table 1: The list of antibodies used in flow cytometry experiments.

Primer name	Sequence / reference number
ALP	Hs01029144_m1
GAPDH	Hs_02758991_g1
NANOG	Hs02387400_g1
OCT4	Hs00999632_g1
PECAM1	Hs00169777_m1
PPARγ	Hs_01115513_m1
RUNX2 Fwd	GACGAGGCAAGAGTTTCACC
RUNX2 Rev	GCCTGGGGTCTGTAATCTGA
SOX2	Hs01053049_s1
TERT	Hs00972650_m1

Supplementary Table 2: The list of primers used in RT-qPCR experiments.
